# Impact of school operating scenarios on COVID-19 transmission under vaccination in the U.S.: an agent-based simulation model

**DOI:** 10.1038/s41598-023-37980-7

**Published:** 2023-08-08

**Authors:** Xingran Weng, Qiushi Chen, Tarun Kumar Sathapathi, Xin Yin, Li Wang

**Affiliations:** 1 Department of Public Health Sciences, A210, Penn State College of Medicine, 90 Hope Drive, Suite 2200, Hershey, PA 17033 USA; 2 Harold and Inge Marcus Department of Industrial and Manufacturing Engineering, Pennsylvania State University, University Park, PA USA

**Keywords:** Infectious diseases, Epidemiology, Viral infection, Epidemiology, Health policy

## Abstract

At the height of the COVID-19 pandemic, K-12 schools struggled to safely operate under the fast-changing pandemic situation. However, little is known about the impact of different school operating scenarios considering the ongoing efforts of vaccination. In this study, we deployed an agent-based simulation model to mimic disease transmission in a mid-sized community consisting of 10,000 households. A total of eight school operating scenarios were simulated, in decreasing order of restrictiveness regarding COVID-19 mitigation measures. When masks were worn at school, work, and community environments, increasing in-person education from 50% to 100% would result in only 1% increase in cumulative infections. When there were no masks nor contact tracing while schools were 100% in person, the cumulative infection increased by 86% compared to the scenario when both masking and contact tracing were in place. In the sensitivity analysis for vaccination efficacy, we found that higher vaccination efficacy was essential in reducing overall infections. Our findings showed that full in-person education was safe, especially when contact tracing, masking, and widespread vaccination were in place. If no masking nor contact tracing was practiced, the transmission would rose dramatically but eventually slow down due to herd immunity.

## Introduction

At the time of our study, more than 45 million people in the US have been infected with COVID-19, with over 740,000 COVID-related deaths by the end of October 2021^1^. At the beginning of the COVID-19 outbreak, in-person education was widely suspended to reduce person-to-person contacts and to slow down the transmission of COVID-19. In the U.S., from March 2020, most states suspended in-person schooling for the 2019–2020 academic year^2–4^.

School closure for an extended time period has brought many social and economic consequences, including impairment of education/early childhood development, psychological harms to children, and loss of free school lunches for eligible children and loss of parental earnings^5–8^. School closure may also indirectly increase COVID mortality, as an estimated 15% of the healthcare workforce was forced to stay home to take care of their kids, which could have led to under-staffing in hospitals and thus more deaths^9^. Many experts in the fields of education and public health have underscored the importance of resuming school-based education for children as soon as possible^10–13^. U.S. President Biden signed a new executive order in January 2021, making the restoration of in-person education a top priority^14^.

To prevent negative consequences caused by school closure, many contingent plans that could allow school-aged children to go back to school have been prepared by policymakers, educators, and public health experts in the U.S.^6,15^ To guide schools to reopen, the U.S. Centers for Disease Control and Prevention (CDC) and/or state governments have recommended flexible instructional modes (e.g., in-person, remote, and hybrid), various risk-level indicators for adjusting instructional modes, and a list of mitigation measures (e.g., facial masks and cleaning)^16–18^. However, resuming full in-person education was a bumpy road. Based on the Household Pulse Survey conducted in October 2020, 65% of households reported that children were learning remotely using online resources^19^. Sporadic COVID-19 outbreaks have forced some schools to close or change the mode of operation since the beginning of the 2020 Fall semester^20–22^. To better protect students and community members, understanding the implications of different school operating scenarios is important to parents, educators, policymakers, and other stakeholders.

To successfully control the pandemic and let students safely return to school, vaccinating more people (adults and children) in the community is critical. As of October 2021, three COVID-19 vaccines have either received full approval (Pfizer/BioNTech) or become available under Emergency Use Authorizations (EUA) by the U.S. Food and Drug Administration (FDA) (Moderna and Johnson & Johnson)^23–26^. As of October 2021, more than 55% of the U.S. population had been fully vaccinated, and a total of about 400 million doses had been administered^27^. The FDA authorized Pfizer/BioNTech’s vaccine for emergency use in children 12 years and older in May 2021, and the FDA further authorized Pfizer’s vaccine for children 5–11 years old in late October 2021^28,29^. In view of the availability of vaccination, the CDC released updated guidelines to assist school districts in providing in-person teaching activities for the fall semester of 2021^30^. At the beginning of Fall 2021, K-12 schools across the country have largely resumed full in-person education^31^.

There are a few simulation studies that have investigated school reopening strategies under the COVID-19 pandemic^32,33^. For instance, Head and colleagues conducted an Agent-Based Simulation (ABS) study to assess various school operating strategies at San Francisco Bay area^32^. One study conducted in Canada used the compartment model to simulate the effects of school reopening on the overall COVID-19 transmission in Toronto^33^. Another study compared the effect of mask vs. no mask in school reopening in Houston, Texas^34^. However, those studies did not consider the effect of increasing vaccination. In addition, those studies were limited to a specific geographic area. Our simulation study would fill this gap by considering the vaccine rollout among school-aged children.

Our study aimed to evaluate the impact of various school operating scenarios on COVID-19 transmission in the context of the vaccine rollout among school-aged children. With the flexible structure of the ABS, we explored the impact of different school operating scenarios on COVID-19 transmission, which can provide much-needed information for school administrators, public health officials, and educators to adapt appropriate safe school operating strategies. During the time of our study, most schools have started to move into partial or full opening. A simulation study like this can shed light on how various risk mitigation measures affect disease transmission at school, and inform policymakers on what public health measures can be employed during a disease outbreak.

## Method

We developed an ABS model to mimic real-world human interactions and resultant COVID-19 transmission, which included four contact networks: home, school, workplace, and community. The ABS has been widely used in epidemiology research to study the dynamics of infectious disease outbreaks^35–37^. It serves as a flexible research tool to characterize complex disease transmission patterns at the individual level^38–41^. ABS has been a popular tool to study transmission of COVID^42–48^. The ABS model we built focused on school reopening, and was developed using Python 3.8.3. The simulation was run through the high-performance computing services provided by the Institute for Computational and Data Sciences at the Pennsylvania State University^49,50^.

### Synthetic population in the agent-based model

Our ABS consisted of 10,000 households with a total of 25,773 individuals. Our virtual population was randomly sampled from a synthetic U.S. population, which has been utilized for populating virtual environments in previous simulation studies^51–54^. Our virtual population was statistically representative of the US population in terms of the distributions of selected variables. For example, the average household size in our simulated population was 2.6 (vs. 2.53 in the US population), and the mean age was 37 years (vs. 38.4 in the US) (For more comparisons, see Appendix Table S1).

Apart from attributes that were inherited directly from the U.S. synthetic population, including age, household index, work status (employed or not), and student status (in K-12 or not), each individual also had the following attributes, including disease state, contact networks, diagnosis status, date of testing, date of diagnosis, quarantine/isolation status, and vaccination status, which were updated during the course of simulation.

### Contact networks

**Figure 1:**
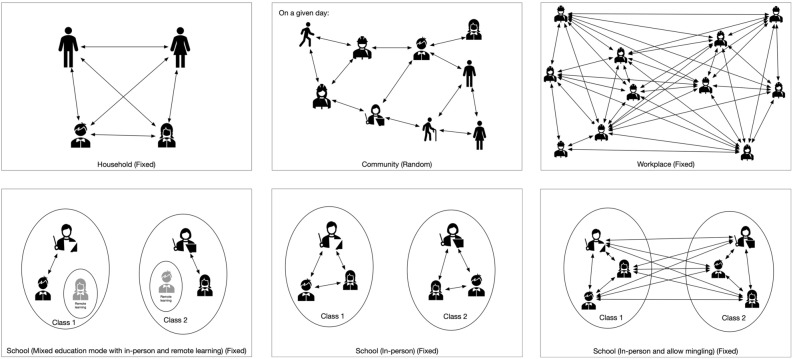
Illustrations of contact networks by environment.

We simulated the disease transmission in different contact networks including household, school, workplace, and community (Fig. 1). For the household networks, individuals with the same household index were in the same household network. We assumed that each household member had contacts with all other members. The household contact network structure remained fixed throughout the entire simulation period.

To construct the school contact networks, we divided all individuals labeled as K-12 students from the synthetic population into multiple smaller networks representing different classes. The size of each class followed a Poisson distribution with a mean class size of 20 students. Similar to existing studies in the literature, we assumed that the class size followed a Poisson distribution, due to the desirable properties of the Poisson distribution to model count data^55^. The class size of 20 in our simulation was similar to the average class size of US public schools reported to be 20.9 in 2017–2018^56^. For each class, we randomly assigned a teacher from the adults whose work status was employed. All students and the teacher of a class were assumed to have contact with one another within the class. In the operating scenarios involving mingling two classes (see the “mingling” scenario in the “School Operating Scenarios” subsection below), all students and the two teachers in the two classes had contact with one another to account for the increased contact.

The workplace contact network involved every working agent in the synthetic population (excluding those assigned as “teachers” earlier). The number of contacts in the workplace network for each individual followed a Poisson distribution with the mean contact number varying by age groups (Appendix Table S2)^57^. Contact networks in the workplace remained fixed throughout the simulation period. In the work environment, it is assumed that workers didn’t switch jobs, everyone worked five days a week unless being quarantined, and there was no distinction in job types, which implied that every job type had the same probability of contacting COVID. Though these simplifications were different from real-world work environment, our model can be easily extended to include more sophisticated assumptions to mimic the real-world situations.

The contact network in the community environment involved all individuals in the synthetic population, and was created in a similar way to the workplace contact network, except that each individual’s community contacts were regenerated every day to represent the randomness of contact in the community (Appendix Table S2)^57^. 

### Model for disease transmission and progression

**Figure 2:**
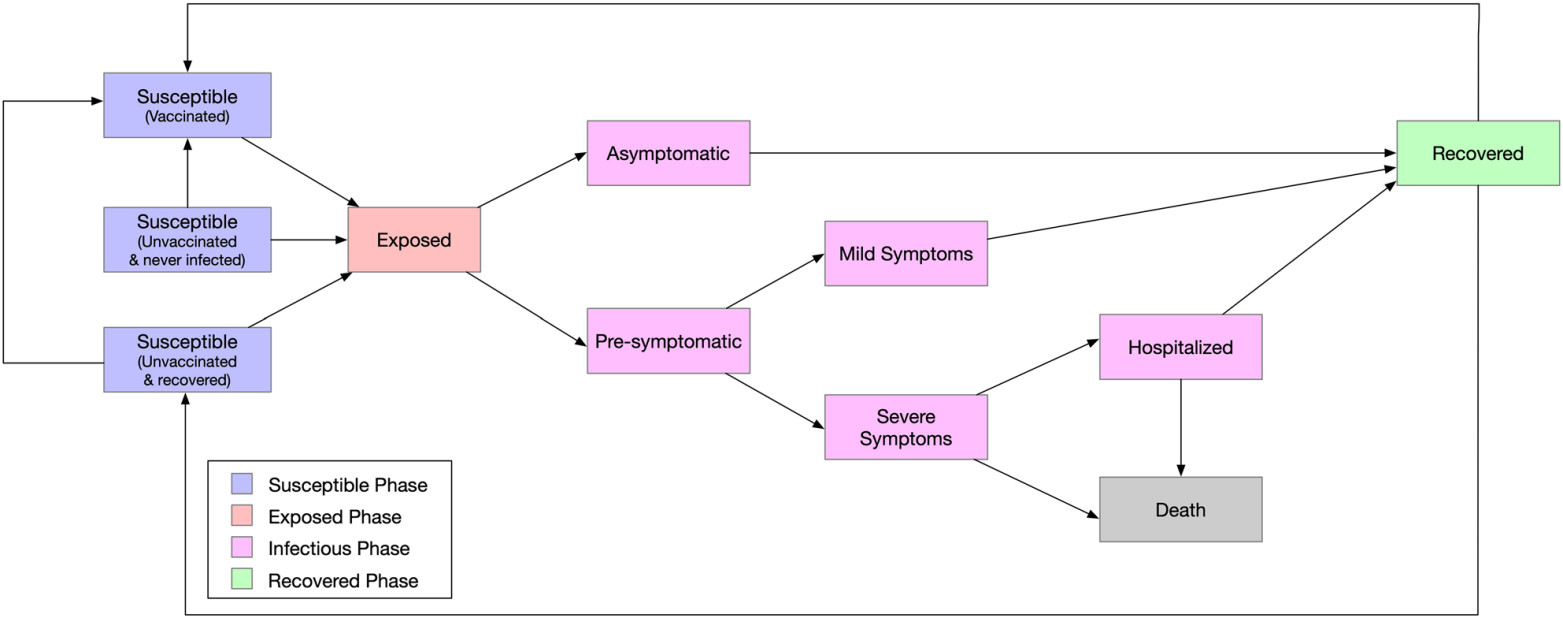
Health state transitions of COVID-19 in the agent-based simulation model.

To simulate the SARS-CoV-2 transmission and the progression of COVID-19, we extended the classic Susceptible-Exposed-Infectious-Recovered (SEIR) model to include vaccination and allow reinfection after recovery (Fig. 2)^58–60^. All individuals were in one of the following mutually exclusive health states on any given day: 1) susceptible, never infected & unvaccinated (i.e., unvaccinated, never infected and susceptible to the disease with a high transmission probability), 2) susceptible & vaccinated (vaccinated and susceptible with a low probability of contracting the disease due to vaccine-induced immunity), 3) susceptible, recovered & unvaccinated (unvaccinated and susceptible with a low probability of contracting the disease due to infection-induced immunity), 4) exposed (infected but not yet infectious), 5) asymptomatic (infectious but will not develop symptoms throughout the course of the disease), 6) pre-symptomatic (infectious but not yet showing symptoms), 7) infected with mild symptoms, 8) infected with severe symptoms, 9) hospitalized, 10) recovered or 11) died as shown in Fig. 2.

Each susceptible individual could be infected by his/her contacts in any network. The baseline transmission probability ($\beta$) was calibrated by running the simulation model under school operation scenario 8 (see explanation of the scenario below) in order to match the estimated basic reproduction number during early outbreak of COVID-19, i.e. ${R}_{0}$=2.2^61^. The probability of transmission was calculated as the baseline transmission probability times an adjustor when applicable. For example, children were assumed to have a lower probability of transmitting the disease, and the transmission probability was reduced by 66% compared to that of adults^62^. The transmission probability was reduced by 50% for asymptotic patients, and recovered individuals had a 84% reduction in the probability of getting the disease^63^. Values of model parameters, probability adjusters and their sources are included in Appendix Table S3.

As shown in Fig. 2, exposed individuals could progress to either the “asymptomatic” state for those who would never show symptoms^64^, or the “pre-symptomatic” state for those who would later progress to a symptomatic state with either mild symptoms or severe symptoms^65^. We differentiated the symptomatic patients by mild and severe cases to represent different clinical pathways. Based on the literature, our model assumed that patients with mild symptoms were diagnosed within at most three days after symptom onset, and then went into home isolation until recovery^65^. Patients showing severe symptoms would be hospitalized^66^. Hospitalized patients would not transmit the disease since they were isolated in the hospital, but these patients were at excess mortality risk due to their critical status. Our simulation model also took into consideration the reinfection risks among recovered individuals^67^. Recovered individuals had a lower risk of being infected again (84% reduction of the transmission probability) due to their acquired immunity from infection^63^. If recovered individuals were later vaccinated, they had 90% reduction in the transmission probability in the baseline analysis.

### Masking, contact tracing, testing, and quarantine/isolation

The following important public health measures were incorporated in our simulation: masking, contact tracing, testing and quarantine/isolation. Wearing facial coverings or masks has proved to be effective against the transmission of COVID-19^68^. Thus, our simulation model considered the protective effect of wearing masks, with a 70% reduction in transmission probability as suggested from empirical evidence^68^.

Contact tracing and timely testing of close contacts of infected patients are essential to effectively control the transmission of COVID-19^69,70^. Once an individual was diagnosed with COVID-19, all other members in the same household and 70% of his/her workplace and school contacts were contact-traced on the same day^71^. People who were contact-traced would be tested on the following day and the test result would also come back on that day. In our model, we did not consider random testing of the population. In comparison, the studies by Asgary et al. and by Panovska-Griffiths et al. have used different testing strategies^72,73^.

Consistent with the CDC’s guidelines, individuals, after showing symptoms or identified as infected, would start home isolation for 14 days, except for those who were hospitalized^74,75^. During home isolation, infected individuals would not transmit the disease through school, workplace, or community networks, but could still transmit the disease within household due to imperfect isolation, for which we assumed a 37% reduction in the transmission probability^70^.

### Vaccination

Given the availability of COVID-19 vaccines and their rapid rollout in the U.S., it is important to consider the impact of vaccination on different school operating strategies^15,16^. For simplicity, our model did not specify the types of vaccine and assumed an overall protection effect of 90% reduction in the transmission probability, based on early evidence on the protective effect of vaccines reflected in the reduction of hospitalization^76,77^.

The model was initialized with a vaccination coverage of 50% among adults and no vaccine for children, which was similar to conditions on May 10th, 2021 when the FDA issued the EUA for 12-to-16-year-old children^78^. The initially vaccinated individuals were randomly sampled from adults in the synthetic population. Our simulation then assigned additional individuals to receive vaccination at a fixed daily vaccination rate. In the analysis, we assumed the baseline daily vaccination rate was equal to vaccinating 0.6% of the adults and 0.5% of the school-aged children, which was similar to the daily administered dosages in the U.S. in June 2021^27^. In view of existing vaccine hesitancy, our model assumed the maximum vaccination coverage of 70% for adults and 40% for children.

In our sensitivity analysis, we also considered lower vaccine efficacies (ranging from 40 to 80%) to account for the reduced protection against emerging variants such as the Delta variant^79^. As the total number of vaccinated U.S. adults started to plateau^27^, our sensitivity analyses assessed different vaccination coverage levels among students (40%, 50%, 60%, and 70%). For fair comparison, the targeted vaccination coverages in sensitivity analysis were reached at the same time (i.e., on day 100) by having different daily vaccination rates. In the sensitivity analysis on student vaccination coverage, the daily vaccination rate for adults remained at 0.6%.

### School operating scenarios

We defined the following eight school operating scenarios. Scenario 1 (*50% alternate in-person*): Students within a class were divided into two groups with group members fixed throughout the simulation, each group having 50% of the students. These two groups alternated between in-person learning and remote learning every school day except on Friday, when all students had remote learning. Scenario 2 (*50% in-person*): 50% of the students received in-person education from Monday to Thursday throughout the simulation period, and the remaining 50% conducted remote learning every school day throughout the period (online education on Friday for all students). Scenario 3 (*80% in-person*): 80% of students received in-person education from Monday to Thursday, while the remaining 20% of students received remote learning throughout the week (online education on Friday for all students). Scenario 4 (*100% in-person*): 100% in-person education for all students from Monday to Friday. In Scenario 5 (*class mingling*), all students had in-person education and mingled with another fixed class for five days a week. Scenario 6 (*No masks at school*) was the same as Scenario 5 except that nobody wore masks in the school environment. Scenario 7 (*No masks anywhere*) further removed masks for workplace and community environments compared to scenario 6. Lastly, Scenario 8 (*No contact tracing*) also discontinued contact tracing on top of Scenario 7. Thus, scenarios 2–8 were in decreasing order of restrictiveness when it came to COVID mitigation measures, scenario 1 and 2 having approximately the same level of restrictiveness. In Scenario 1 through Scenario 4, since no mingling was allowed, there was no teacher interaction as well. In Scenario 5 through Scenario 8, where two classes mingled, both the students and the teachers in those classes mingled. Since high school students tend to have more inter-class interaction than elementary school students, Scenario 5 would be a more realistic model for high schools than scenario 4.

### Simulation analysis and outcomes

As it was already more than one year into the pandemic at the time of our study, we did not simulate the scenarios from the beginning. Instead, we used a warm-up period to initialize the model by simulating scenario 8 until it reached the cumulative infection rate of approximately 10%, which was comparable to the infection rate in early June 2021 in the U.S^80^. The warm-up period started with 25 exposed cases who were randomly sampled from the synthetic population (comparable to approximately 0.1% of the total population being infected at the beginning of April 2020 in the U.S.)^81^. After the initialization, a total of 250 days –-approximately equal to the duration of an academic year–- was simulated to evaluate the model outcomes for each school operating scenario.

The primary model outcomes included the numbers of new daily cases and cumulative infections. The 7-day moving averages were used to produce smoother estimates of daily cases, so the first reported data point for the daily new cases was on day 7 after model initialization. Cumulative infections were reported for all environments together and stratified by household, school, workplace, and community. To quantify the uncertainty of stochastic simulation outcomes, we ran each scenario with 200 independent replications and reported the mean values and the 95% confidence intervals (mean ± 1.96*standard error)^82^. Lastly, we performed sensitivity analyses on student total vaccination coverage and vaccine efficacy.

## Results

### COVID-19 Infections under Different School Operating Scenarios

**Figure 3:**
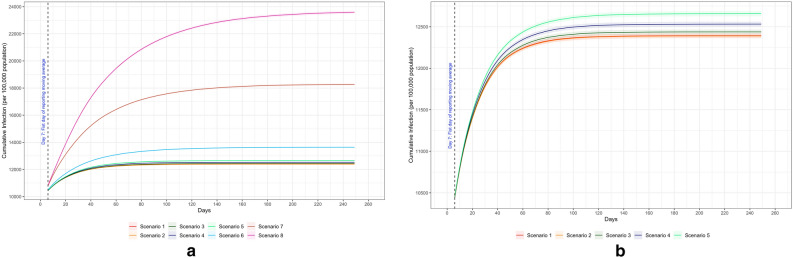
Cumulative Infections of COVID-19 by school operating scenarios.

Figure 3 shows the cumulative infection curves for all scenarios, with Fig. 3.b being a close-up view of Scenarios 1–5, and Table 1 shows the exact numbers of cumulative infections and daily infections, with two data points highlighted for daily infections: the first and the last data point. Scenarios 1 and 2 represented the two most restrictive scenarios that differed only by alternating or fixing the in-person and remote learning groups within each class. As shown in Table 1, these two scenarios resulted in the lowest infection rates. Scenario 1 and 2 had nearly identical cumulative infection rates (both rounded to 12.4%, 95% CI for Scenario 1: 12.37–12.42% and 95% CI for Scenario 2: 12.36–12.42%). The cumulative infections did not increase much with a higher percentage of in-person learning when public health mitigation measures were in place, being 12.4% (95% CI: 12.41%—12.47%) for 80% in-person learning in Scenario 3 and 12.5% (95% CI 12.50–12.56%) for 100% in-person learning in Scenario 4.

**Table 1:** Daily incidence and cumulative infection for each school operating scenario.

Scenario # *(Short name)*	Cumulative infection^a^		Incidence^a^	
	Total (95% CI)^b^	Percentage (%)	First 7-day average (95% CI)^b^	Last 7-day average (95%CI)^b^
1 (50% alternate in-person)	12,392.6 (12,365.2, 12,420.0)	12.4	161.6 (159.8, 163.4)	0 (0, 0)
2 (50% in-person)	12,388.7 (12,360.6, 12,416.9)	12.4	161.7 (159.9, 163.5)	0 (0, 0)
3 (80% in-person)	12,437.9 (12,409.1, 12,466.7)	12.4	161.5 (159.7, 163.2)	0.01 (0, 0.01)
4 (100% in-person)	12,532.3 (12,502.9, 12,561.6)	12.5	164.4 (162.7, 166.2)	0.01 (0, 0.01)
5 (Class mingling)	12,657.9 (12,626.7, 12,689.1)	12.7	165.5 (163.8, 167.3)	0.01 (0, 0.01)
6 (No masks at school)	13,633.2 (13,589.5, 13,676.9)	13.6	177.5 (175.8, 179.2)	0.06 (0.02, 0.09)
7 (No masks anywhere)	18,272.4 (18,194.9, 18,350.0)	18.3	303.1 (300.7, 305.5)	0.48 (0.37, 0.58)
8 (No contact tracing)	23,593.6 (23,490.7, 23,696.4)	23.6	323.0 (320.3, 325.6)	2.09 (1.84, 2.33)

^a^Both incidences and cumulative infections reported in the table were per 100,000 population.

^b^Confidence intervals were constructed based on the standard error of 200 replications.

Allowing mingling in school (Scenario 5) had a marginal effect on the total infections, with an increase of 0.2 percentage points (12.7%, 95% CI 12.63–12.69%). When mask wearing was removed from schools in Scenario 6, the cumulative infection increased to 13.6% (95% CI 13.59–13.68%). When neither the work nor community environments had masks in Scenario 7, the cumulative infection rate increased substantially to 18.3% (95% CI 18.19–18.35%). By further removing contact tracing in Scenario 8, which was the least restrictive scenario, we estimated that the cumulative infection rate would reach 23.6% (95% CI 23.49%–23.70%). Results from Scenario 6 through 8 showed that by eliminating mask wearing and contact tracing from the beginning, cumulative infections could increase drastically.

**Figure 4:**
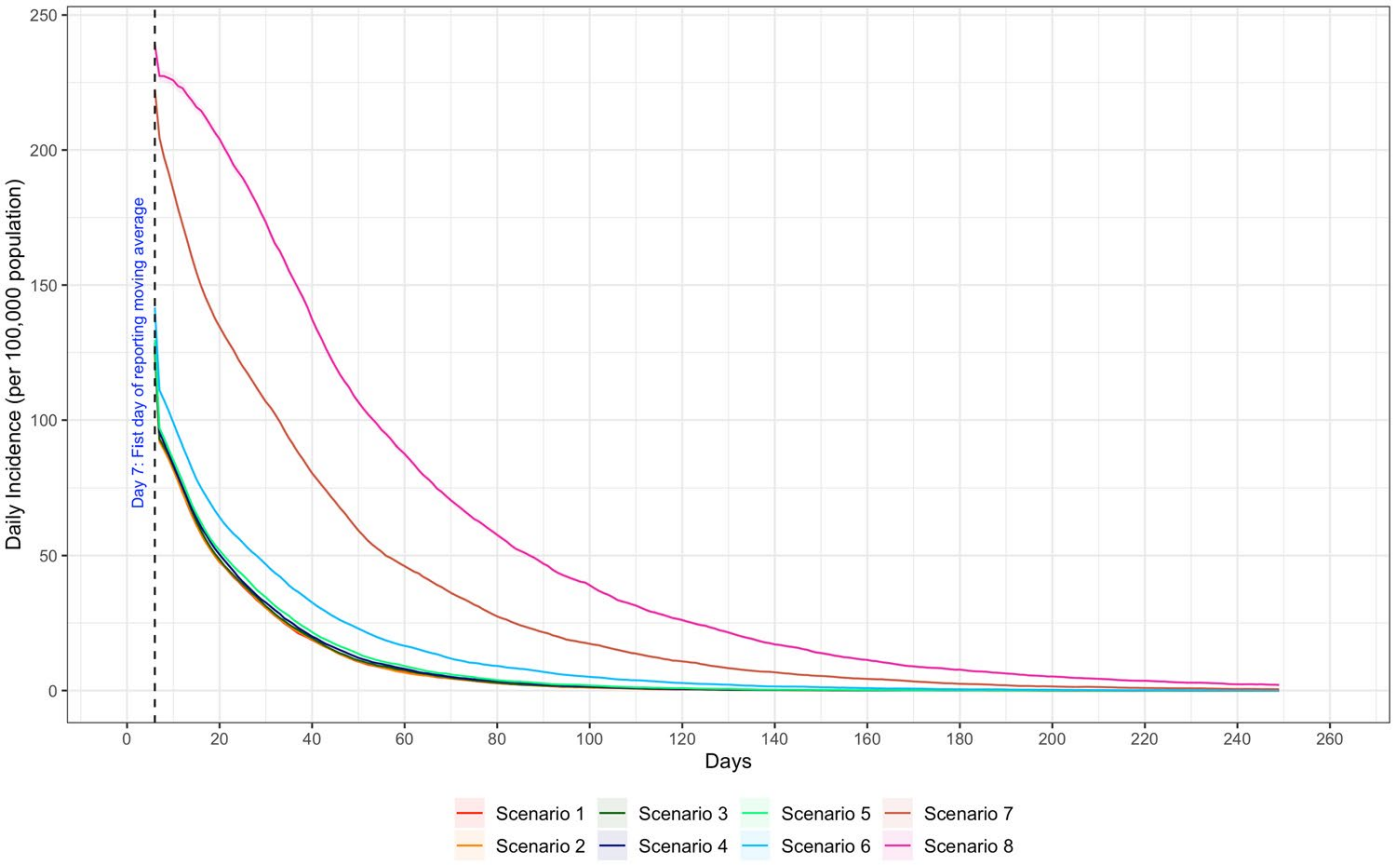
Daily Incidence by school operating scenario.

Figure 4 shows the 7-day moving average of daily new infections for each scenario. All scenarios showed a decreasing trend in disease transmission due to increasing immunity in the population (note that the model did not introduce factors that would cause an increase in infections over the course of simulation, such as holiday gatherings). Similar to cumulative infections, the new daily incidences in Scenarios 6–8 were significantly higher than those in Scenarios 1–5 throughout the simulation. As shown in Fig. 4, it took Scenario 1–5 about 20 days to cut its daily incidences down to 50 people per 100,000 population, while in Scenarios 6–8, it took approximately 30 days, 60 days, and 90 days, respectively (Fig. 4). Towards the end of the simulation period (the last 7-day moving average), the transmission was extremely low, with almost 0 case in Scenario 1 through 5.

### Cumulative COVID-19 Infections by Environment

**Figure 5:**
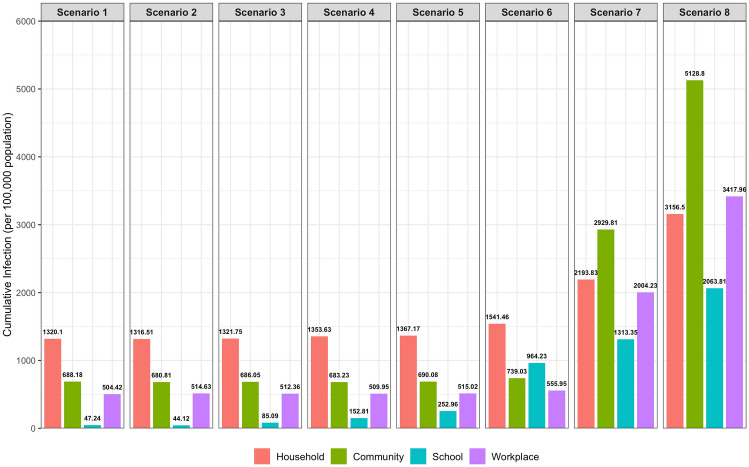
Total post-warmup infections by environment- for each school operation scenario.

Figure 5 shows the total post-warmup infections (not counting the cumulative infection rate of 10% during the warm-up period) by environment for each school operating scenario. With the increase in the percentage of in-person education from 50%  to 100% (Scenario 2 through 4), the infections contracted in the school environment increased from about 44 (Scenario 2) to 153 (Scenario 4) per 100,000 population. We did not observe substantial “spill-over” effect in increasing new infections in household, workplace, or community environment. Compared to Scenario 1 (50% alternate in-person), Scenario 4 (100% in-person) saw an increase of about 34 cases per 100,000 population in the household environment, six cases in the community, and three cases in workplace, much lower than the increase of 120 cases in the school environment. Similar lack of “spill-over” effect in non-school environments was found in Scenario 5, which allowed mingling in school. In Scenario 6, eliminating mask wearing at school resulted in a substantial increase in the total post-warmup infections only in the school environment, but not much “spill-over” effects were observed in non-school environments (workplace, household, and community) since mask-wearing still was required in those places.

When masks were no longer worn in any environment (Scenario 7 and 8), the total post-warmup infections increased substantially in all environments. In Scenario 7, the total post-warmup infections from both community and workplace saw about 296% and 260% increases respectively compared to Scenario 6. The increase of infections was 42% in households and 36% in schools. In Scenario 8, the cumulative post-warmup infections were the highest among all scenarios in each of the four environments. Furthermore, the largest proportion of post-warmup cumulative infections occurred in the community environment in Scenario 8 (37%).

### Effects of Vaccine Parameters on COVID-19 infections

To assess the impact of vaccination on the overall COVID-19 transmission, we conducted sensitivity analyses on the maximum vaccination coverage among students and the vaccine efficacy, using Scenario 7 as an example.

**Figure 6:**
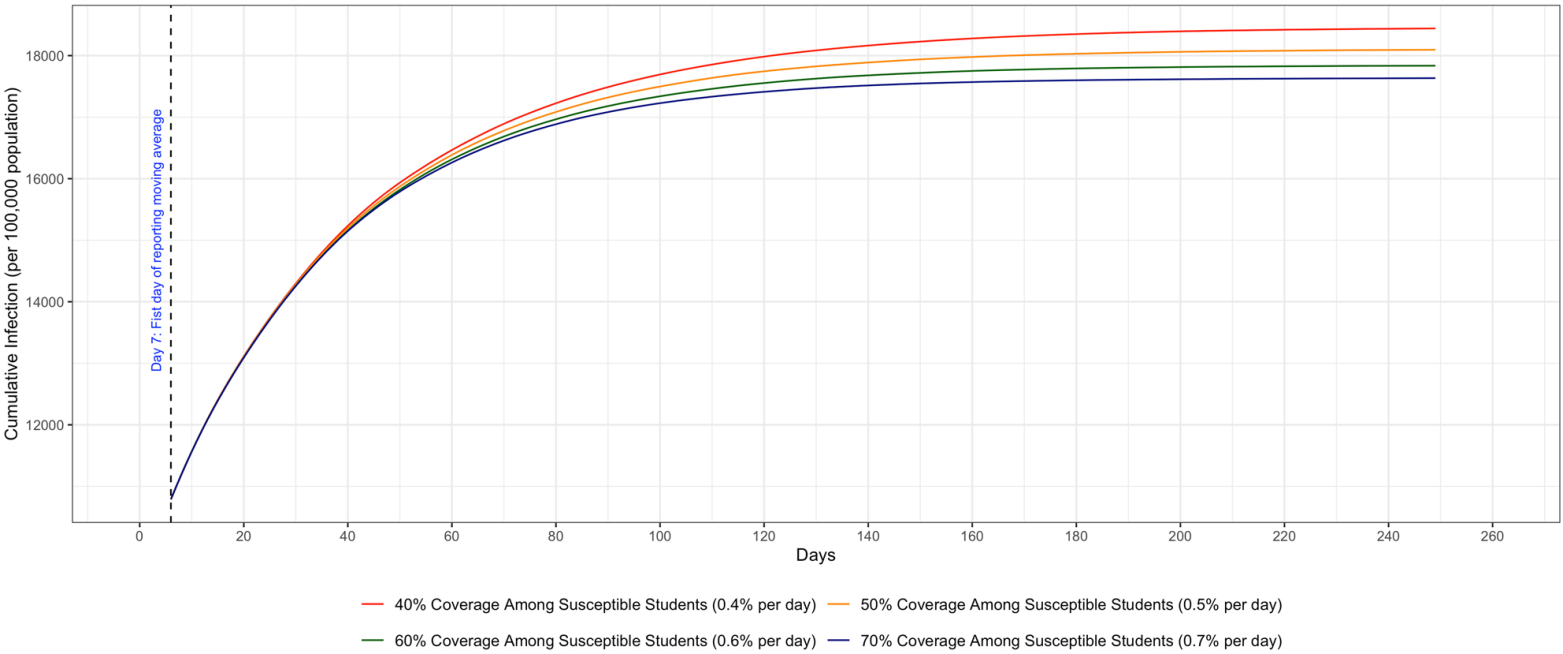
Different vaccination coverage among students (40%, 50%, 60%, and 70%).

To assess various maximum vaccination coverages (from 40% to 70%) among students, different daily vaccination rates were used to ensure all targeted coverage percentages were reached at about the same time (day 100). As shown in Fig. 6, the highest vaccination coverage (70%) with 0.7% daily vaccination rate among students led to the lowest cumulative infections on Day 250 (17,634.3 people per 100,000 population, 95% CI: 17,563.6–17,705.1), compared to the lowest vaccination coverage of 40% reached by a daily vaccination rate of 0.4% (18,441.8 people per 100,000 population, 95% CI 18,363.2–18,520.5). We found that a higher vaccination coverage (through a higher daily vaccination rate) in the student population reduced cumulative infections.

**Figure 7:**
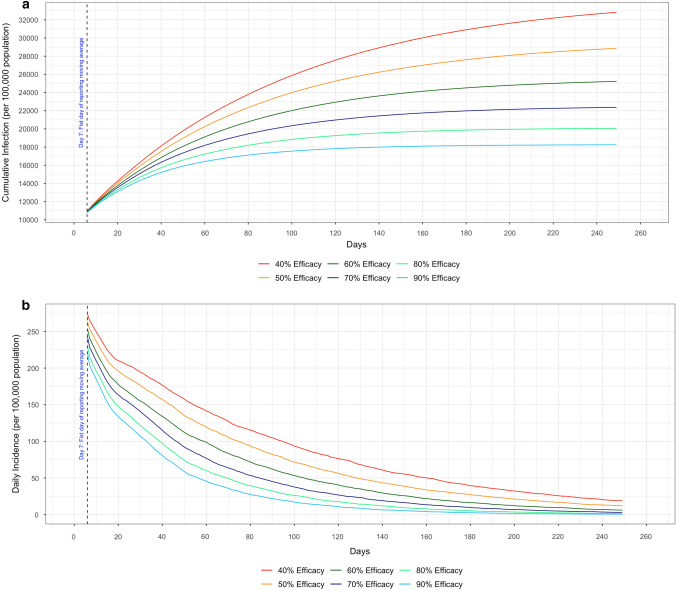
Different vaccine efficacy (40%, 50%, 60%, 70%, 80% and 90%).

Additionally, six different vaccine efficacies were evaluated to account for circumstances where the vaccine may have lower efficacy. According to the cumulative infection curves in Fig. 7, if the vaccine efficacy dropped to 40%, the cumulative infections on Day 250 could climb to as high as 33% of all population (32,828.5 people per 100,000 population, 95% CI 32,646.5–33,010.5). As the efficacy increased, the total infections were significantly reduced. With the highest efficacy (90%), an additional 15% of the population would avoid getting infected when compared to the efficacy of 40%. As shown in the daily incidence curves in Fig. 7, the lowest vaccine efficacy (40%) took 160 days to reduce to a daily infection rate of 50 people per 100,000 population. However, the highest vaccine efficacy (90%) declined to the same level of infection within 60 days, which was about 100 days earlier.

## Discussion

This agent-based simulation study assessed different school operating scenarios while factoring in vaccinations. Based on the simulation, COVID-19 mitigation measures still play a key role in reducing the transmission, even while the vaccination coverage increases. Our results indicated that if mitigation measures were relaxed prematurely, schools and communities would observe significantly higher cumulative COVID-19 infections, despite widespread vaccination. Sensitivity analyses showed that high vaccination coverage and strong vaccine efficacy were crucial factors in ensuring optimal effects of vaccination. One takeaway from this study is that public health mitigation measures can’t be relaxed prematurely even with increasing vaccination, in order to ensure safer school operation.

The models saw monotonically decreasing daily new infections due to increased immunity in the population either through infection or vaccination. By day 250 in our model, the pandemic had ended due to herd immunity, as the new daily infections nearly approached zero. In reality, the pandemic may not end as thoroughly and soon as in our model, because there are numerous changing factors that affect the dynamics of the real-world pandemic. The emergence of new variants and the changes in people’s behaviors such as increasing of social gatherings could introduce new disease transmission dynamics into the pandemic. Those changing factors were part of the reasons why peaks and troughs were seen in the real-world disease trends. Our simulation, instead of addressing numerous dynamic factors in the real-world, focused on the effects of different school operating scenarios, while keeping other factors fixed. In particular, the whole simulation was run considering only one COVID-19 strain of fixed transmissibility and toxicity, with no changes in people’s behaviors within a given scenario.

With regards to COVID-19 variants, our simulation model currently only considered the original strain. However, as of July 2021, there were at least 10 variants of SARS-CoV-2 circulating, and the B.1.617.2 variant (Delta variant) has become the dominate variant in the U.S.^83^ The mutations of SARS-CoV-2 have imposed unpredictable and serious challenges to curbing the COVID-19 pandemic^84,85^. Based on emerging findings, both the B.1.1.7 (Alpha variant) and Delta variants are more likely to transmit to susceptible individuals^86,87^, cause more serious symptoms^87^, reduce the effectiveness of anti-viral medications^88,89^, and lead to lower efficacy of vaccines^90,91^. Though our model did not consider variants, it can be used to study the impacts of variants by changing certain model parameters, such as the efficacy of the vaccine and the probability of transmitting the disease. Based on testing different vaccine efficacies (Fig. 5), our analyses demonstrated that a lower efficacy of the vaccine would result in a spike in infections. For instance, if the vaccine efficacy was reduced from 90 to 60% while holding other factors of the model fixed, the cumulative infection rate on Day 250 rose from 18% to more than 22% in the case of Scenario 7. This sharp increase emphasized the potential outbreak risks of new variants, if the vaccine could not deliver the same high efficacy as for the original virus strain. Moreover, our model can be modified (albeit substantially), to assess the transmission dynamics when multiple COVID variants are circulating at the same time.

Our model only included a hypothetical type of one-dose vaccine that immediately took effect after inoculation, without considering the necessity of a second shot or a two-week wait period to be fully vaccinated^76^. The simplification to one vaccine, one shot and immediate effectiveness in our model would not affect the study’s overall conclusions, for it essentially moved the timeline ahead by about one month with all the transmission dynamics preserved. By focusing on the essential features of the vaccination effect, our current simulation model demonstrates that vaccination is a key factor to control the pandemic and safely operate schools.

Our study underscored that wide vaccination coverage among children is an important element to successfully control the pandemic. Though children are less likely to be infected, scaling up their vaccination coverage could effectively prevent them from passing the virus to high risk adults in the household, school, and community environments^92^. According to the analyses in the result section (Fig. 6), raising the vaccination coverage from 40% to 70% among students resulted in a reduction of more than 4 percentage points in cumulative infections, when adults’ maximal vaccination coverage was kept at 70% (6 percentage points reduction if the vaccination coverage was kept at 40% for adults). This suggested that, in some communities with low adult vaccination coverage, having more students vaccinated could effectively reduce community transmission. To achieve the optimal level of population immunity (75– 80% of the entire U.S. population), it is important for parents to have their children vaccinated. Nevertheless, considering that as many as one fifth of parents in the U.S. have influenza vaccine hesitancy, parents may be reluctant to give their children COVID-19 vaccines^93^. Therefore, our simulation model employed a parameter describing the maximal vaccination coverage for students, which can be modified to reflect different levels of vaccine hesitancy.

As for the school environment, the current model did not differentiate among primary, middle and high schools, whereas the study conducted in San Francisco did so^32^. By allowing more classes to mingle instead of just two in our model, our simulation can potentially mimic the larger contact network found in high schools. However, given that students aged 12 and above were eligible for vaccines months before those aged 5–11, differentiating school levels would create complex implications to the model such as the determination of which child got the vaccine at what point in time, and unnecessarily complicate the interpretation of the results. The simplification of not differentiating schools would not change the directions of overall conclusions on the necessity of vaccination and mitigation measures.

Our simulation results showed that masking significantly reduced the number of infections, and was a key factor in limiting disease transmission. In our model, masking was assumed to reduce the probability of transmission by 70%, which was consistent with the estimated effectiveness of masks in the literature^94,95^. Our simulation model assumed either no masking for anyone or universal masking for everyone in different scenarios to explore the effect of masking. Partial masking can be assessed by assuming that a certain percentage of people would still wear masks when masking is not required, while a proportion of people would not wear masks even though masking is required. Another assumption in our model was that when masking was required, everyone irrespective of vaccination status needed to be masked. According to the CDC’s recommendation, people who are fully vaccinated can be unmasked in certain cercumstances^76^. However, our model’s assumption of universal masking would not affect the directions of our overall conclusions, for the effect of masks in our model was mainly through masking unvaccinated people since those vaccinated already had 90% protection from vaccines. The guidance for safe opening schools recently prepared by the American Academy of Pediatrics (AAP) recommended a universal masking policy when resuming full capacity in-person education^96^. It is difficult to strictly implement a selective masking requirement excluding those vaccinated, and universal masking can minimize the possibility of contracting the virus at school.

Our study has a few limitations. Firstly, for regions of the U.S. with very dissimilar demographic characteristics to the synthetic population used in this study, the simulation results may not be generalizable. The synthetic population was generated based on the U.S. census^51^; therefore, interpretations of the results for other countries require caution. Secondly, as an agent-based microsimulation model to mimic the reality, our study may have oversimplified some aspects of the reality. For instance, our model did not consider the possibility of increasing transmissibility before symptoms showed up, nor did it consider waning vaccination effect over time. Thirdly, this study did not consider factors that could change in the midst of the simulation, such as changing human behaviors. For example, people may get COVID-19 fatigue and let their guard down. Future models can incorporate changing human behaviors to model how they affect the trajectory of the pandemic. Lastly, the size of the synthetic population used in this simulation model was only around 25,000 due to computing power. However, it was based on the U.S. census data and captured the essential features of what a larger U.S. population would be like. Therefore, the results should also be informative for areas with a population size much larger than the one in our study.

## Conclusion

This simulation study evaluated multiple school operating scenarios based on an agent-based simulation model while accounting for vaccination efforts. Our findings highlighted the importance of COVID-19 mitigation measures (e.g., masking and contact tracing) for schools to safely operate, even with increasing vaccination coverage. Our model shows that with public health measures and widespread vaccination, safely operating schools with 100% in-person learning is feasible and does not cause substantial increase of infections. If no masking nor contact tracing was practiced, the transmission would rose dramatically but eventually slow down due to herd immunity from both infection and vaccination.

### Supplementary Information

Supplementary Information.

## Data Availability

The simulated datasets are available from the corresponding author upon request.
