# Supplementary material for: Impact of school operating scenarios on COVID-19 transmission under vaccination in the U.S.: an agent-based simulation model
Source: Sci Rep. 2023 Aug 8;13:12836. doi: 10.1038/s41598-023-37980-7 (PMC10409779; doi:10.1038/s41598-023-37980-7)
Supplement: Supplementary file 1 — Supplementary Information. [file 41598_2023_37980_MOESM1_ESM.docx]

**Supplementary Document:**

**Impact of School Operating Scenarios on COVID-19 Transmission Under Vaccination in the U.S.: An Agent Based Simulation Model**

**Author List:**

Xingran Weng^1^, Qiushi Chen^2^, Tarun Kumar Sathapathi^2^, Xin Yin^1^, Li Wang^1^

**Affiliations:**

1. Department of Public Health Sciences, Penn State College of Medicine, Hershey, Pennsylvania, USA
2. Harold and Inge Marcus Department of Industrial and Manufacturing Engineering, Pennsylvania State University, University Park, Pennsylvania, USA

**Corresponding Author:**

Li Wang, PhD

Associate Professor

Department of Public Health Sciences, A210

Penn State College of Medicine

90 Hope Drive, Suite 2200

Hershey, PA 17033

Tel: 717-531-5949

Fax: 717-531-5779

Email: [luw119@psu.edu](mailto:luw119@psu.edu)

**Appendix Table S1: Demographic characteristics of the virtual population in the ABS compared with those of the U.S. population**

| **Demographic Characteristics** | | **population in the ABS (N=25,773)** | **U.S.**  **population** |
| --- | --- | --- | --- |
| Average Household Size | | 2.6 | 2.53 ^1^ |
|  | School-aged individuals ($\leq18$ years old) (%) | 5,203 (20.2%) | 24.6% ^2^ |
|  | Working individuals (%) | 11,935 (46.3%) | 47.8% ^3^ |
| Mean age | | 37 | 38.4 ^4^ |
|  | Male | 12,603 (48.9%) | 49.2% ^5^ |
|  | Female | 13,170 (51.1%) | 50.8% ^5^ |

**Appendix Table S2: Daily number of contacts by environment in different age groups**

| **Environment** | **Daily Contacts** |
| --- | --- |
| Workplace | Less than 19 years old: 10/day  20-69 years old: 7/day  70 and older: 3/day |
| Community | Less than 19 years old: 2/day  20-69 years old: 4/day  70 and older: 3/day |

**Appendix Table S3:** **Parameters used in the simulation and their sources**

| **Notation** | **Parameter** | **Value** | **References** |
| --- | --- | --- | --- |
| **Disease Natural History Parameters** | | | |
| $D_{q}$ | Duration of quarantine | 14 days | CDC^6^ |
| $D_{E}$ | Duration of exposed state | 4 days | Davies et al.^7^ |
| $D_{A}$ | Duration of asymptomatic state | Median: 18 days  Range 5 to 28 | Yongchen et al.^8^ |
| $\rho$ | Probability of being asymptomatic | 0.45 | Oran, Topol^9^ |
| $\delta$ | Probability of severe symptoms | 19% | Wu, McGoogan^10^ |
| $D_{M}$ | Duration of mild symptom state | Median: 10 days  Range 2 to 21 | Yongchen et al.^8^ |
| $D_{S}$ | Time from severe symptom to hospitalization | Median 1.2 days  Range 0.2 to 29.9 | Lauer et al.^11^ |
| $D_{H}$ | Hospitalization duration | 7 days (interquartile range 3-13; full range 0-52 days) | Petrilli et al.^12^ |
|  | Daily probability of transition between states | To convert from the duration in a given state of $d$ days into the daily transition probability between two states, we assumed exponential distribution for the transition time, resulting in the transition probability per day as $p(d)=1-\exp\left( -\frac{1}{d} \right)$. |  |
| $\mu$ | Daily mortality rate of severe cases | A fatality rate of 0.00144/day | The Novel Coronavirus Pneumonia Emergency Response Epidemiology Team ^13^ |
| $\gamma$ | Relative Susceptibility of COVID-19 among children | 66% reduction | Zhang et al.^14^  Dattner et al.^15^ |
| **Disease Transmission Parameters** | | | |
| $\beta$ | Baseline transmission probability | calibrated from the model based on the R0 in the literature | Li et al.^16^ |
| $\alpha_{r}$ | Reduction of transmission probability among recovered individuals | 84% | Hall et al.^17^ |
| $\alpha_{h}$ | Relative reduction of transmission probability by isolation | 37% reduction of transmission probability in household for self-isolation | Kucharski et al.^18^ |
| $\alpha_{m}$ | Relative reduction of transmission probability by wearing mask | 70% reduction of transmission probability | Howard et al.^19^ |
| $\alpha_{a}$ | Relative reduction of transmission probability in asymptomatic cases | 50% | Davies et al.^7^ |
| **Vaccine Parameters** | | | |
| $\alpha_{v}$ | Vaccine efficacy: relative reduction in transmission probability if vaccinated | 90% (simplified assumption based on all three authorized vaccines) | Moline et al.^20^ |
| $V_{a}$ | Baseline daily vaccination rate for adults | 0.6% among adults calculated based on data in June, 2021 | CDC^21^ |
| $V_{c}$ | Baseline daily vaccination rate for children | 0.5% among students calculated based on data in June, 2021 | CDC^21^ |

**References**

1. U.S. Census Bureau. Table HH-4. Households by Size: 1960 to Present. 2020; <https://www2.census.gov/programs-surveys/demo/tables/families/time-series/households/hh4.xls>. Accessed Feb 1st, 2021.

2. U.S. Census Bureau. Table 1. Enrollment Status of the Population 3 Years Old and Over, by Sex, Age, Race, Hispanic Origin, Foreign Born, and Foreign-Born Parentage: October 2018. 2019; <https://www2.census.gov/programs-surveys/demo/tables/school-enrollment/2018/2018-cps/tab01-01.xlsx>. Accessed Feb 1st, 2021.

3. Statista.com. Employment in the United States 2021. 2021; <https://www.statista.com/statistics/269959/employment-in-the-united-states/>. Accessed Feb 1st, 2021.

4. Statista.com. Median age of the U.S. population 1960-2019. 2021; <https://www.statista.com/statistics/241494/median-age-of-the-us-population/>. Accessed Feb 1st, 2021.

5. Bureau USC. Age and Sex Composition: 2010. 2010; <https://www.census.gov/prod/cen2010/briefs/c2010br-03.pdf>. Accessed Feb 1st, 2021.

6. Centers for Disease Control and Prevention. Interim Guidance on Duration of Isolation and Precautions for Adults with COVID-19. 2021; <https://www.cdc.gov/coronavirus/2019-ncov/hcp/duration-isolation.html>. Accessed March 20th, 2021.

7. Davies NG, Kucharski AJ, Eggo RM, et al. Effects of non-pharmaceutical interventions on COVID-19 cases, deaths, and demand for hospital services in the UK: a modelling study. *The Lancet Public Health.* 2020;5(7):e375-e385.

8. Yongchen Z, Shen H, Wang X, et al. Different longitudinal patterns of nucleic acid and serology testing results based on disease severity of COVID-19 patients. *Emerg Microbes Infect.* 2020;9(1):833-836.

9. Oran DP, Topol EJ. Prevalence of Asymptomatic SARS-CoV-2 Infection : A Narrative Review. *Ann Intern Med.* 2020;173(5):362-367.

10. Wu Z, McGoogan JM. Characteristics of and Important Lessons From the Coronavirus Disease 2019 (COVID-19) Outbreak in China: Summary of a Report of 72314 Cases From the Chinese Center for Disease Control and Prevention. *JAMA.* 2020;323(13):1239-1242.

11. Lauer SA, Grantz KH, Bi Q, et al. The Incubation Period of Coronavirus Disease 2019 (COVID-19) From Publicly Reported Confirmed Cases: Estimation and Application. *Ann Intern Med.* 2020;172(9):577-582.

12. Petrilli CM, Jones SA, Yang J, et al. Factors associated with hospital admission and critical illness among 5279 people with coronavirus disease 2019 in New York City: prospective cohort study. *BMJ.* 2020;369:m1966.

13. The Novel Coronavirus Pneumonia Emergency Response Epidemiology Team. The Epidemiological Characteristics of an Outbreak of 2019 Novel Coronavirus Diseases (COVID-19) — China, 2020. *China CDC Weekly.* 2020;2(8):113-122.

14. Zhang J, Litvinova M, Liang Y, et al. Changes in contact patterns shape the dynamics of the COVID-19 outbreak in China. *Science.* 2020;368(6498):1481-1486.

15. Dattner I, Goldberg Y, Katriel G, et al. The role of children in the spread of COVID-19: Using household data from Bnei Brak, Israel, to estimate the relative susceptibility and infectivity of children. *PLoS Comput Biol.* 2021;17(2):e1008559.

16. Li Q, Guan X, Wu P, et al. Early Transmission Dynamics in Wuhan, China, of Novel Coronavirus-Infected Pneumonia. *N Engl J Med.* 2020;382(13):1199-1207.

17. Hall VJ, Foulkes S, Charlett A, et al. SARS-CoV-2 infection rates of antibody-positive compared with antibody-negative health-care workers in England: a large, multicentre, prospective cohort study (SIREN). *The Lancet.* 2021;397(10283):1459-1469.

18. Kucharski AJ, Klepac P, Conlan AJK, et al. Effectiveness of isolation, testing, contact tracing, and physical distancing on reducing transmission of SARS-CoV-2 in different settings: a mathematical modelling study. *The Lancet Infectious Diseases.* 2020;20(10):1151-1160.

19. Howard J, Huang A, Li Z, et al. An evidence review of face masks against COVID-19. *Proceedings of the National Academy of Sciences of the United States of America.* 2021;118(4).

20. Moline HL, Whitaker M, Deng L, et al. Effectiveness of COVID-19 Vaccines in Preventing Hospitalization Among Adults Aged ≥65 Years — COVID-NET, 13 States, February–April 2021. *MMWR Morbidity and Mortality Weekly Report.* 2021;70(32).

21. Centers for Disease Control and Prevention. COVID-19 Vaccinations in the United States. 2021; <https://covid.cdc.gov/covid-data-tracker/#vaccinations>. Accessed June 5th, 2021.
